# Possible Recurrent Pandemic (H1N1) 2009 Infection, Israel

**DOI:** 10.3201/eid1608.100436

**Published:** 2010-08

**Authors:** Eran Kopel, Michal Mandelboim, Ziva Amitai, Itamar Grotto, Musa Hindiyeh, Ehud Kaliner, Ella Mendelson, Irina Volovik

**Affiliations:** Ministry of Health, Tel Aviv, Israel (E. Kopel, Z. Amitai, E. Kaliner, I. Volovik); Ministry of Health, Ramat Gan, Israel (M. Mandelboim, M. Hindiyeh, E. Mendelson); Ministry of Health, Jerusalem, Israel (I. Grotto); 1 These authors contributed equally to this article.

**Keywords:** Influenza A virus, H1N1 subtype, reinfection, relapse, case report, viruses, Israel, letter

**To the Editor:** We report 2 cases of possible recurrent laboratory-confirmed infection with pandemic (H1N1) 2009 virus in Israel. Patient 1, a 24-year-old man, had Noonan syndrome (*1**,**2*). He was hospitalized on August 10, 2009, because of high-grade fever and cough. At admission, a nasopharyngeal specimen was collected for pandemic (H1N1) 2009 virus real-time reverse transcription–PCR (RT-PCR) (ABI 7500; Applied Biosystems, Foster City, CA, USA) for the pandemic hemagglutinin gene; a validated in-house protocol developed at Israel Central Virology Laboratory was used, as previously described (*3*). Briefly, the in-house assay was validated against the assay for pandemic (H1N1) 2009 virus developed by the Centers for Disease Control and Prevention (CDC; Atlanta, GA, USA). The in-house assay was as sensitive as the CDC assay; however, the in-house primers and probes were more specific for detecting pandemic (H1N1) 2009 virus with 105% amplification efficiency of viral RNA that was logarithmically serially diluted. In addition, of 100 samples tested side by side with the in-house and CDC assays, 75 samples were positive by both assays, and 25 were negative by both assays; thus, the sensitivity and specificity of the in-house assay were 100%.

The patient was not treated with neuraminidase inhibitors and did not require supportive treatment; after 1 day of hospitalization, he was discharged with a diagnosis of upper respiratory tract infection. The laboratory subsequently reported the RT-PCR as positive for pandemic (H1N1) 2009 virus. On November 22, the man was hospitalized again for dyspnea and fever. The RT-PCR result from a nasopharyngeal sample collected at admission was positive. Hemagglutination-inhibition assay demonstrated a high titer (320) of serum antibody against pandemic (H1N1) 2009 virus in a blood sample taken at admission. The patient took oseltamivir for 5 days, and his condition markedly improved. Result of a repeat RT-PCR at discharge was negative.

An identical neuraminidase gene sequence was detected during both illness episodes (August and November). The specimens were also tested with an experimental RT-PCR assay for rapid detection of the oseltamivir resistance mutation H275Y on the pandemic neuraminidase gene (*4*). For specimens collected during both episodes, the virus was oseltamivir sensitive.

Patient 2, a 13.5-year-old boy, had severe cerebral palsy. On July 27, 2009, high-grade fever with dyspnea developed. He was treated as an outpatient for 5 days with oseltamivir and clinically improved. However, on August 11, he had fever with respiratory distress and was hospitalized. RT-PCR for pandemic (H1N1) 2009 virus was positive on August 14. A second course of oseltamivir was administered for 10 days with the dosage adjusted for age and doubled from that of the previous regimen. Further testing with the experimental rapid RT-PCR indicated the viral strain had the oseltamivir resistance mutation. On September 14, RT-PCR was negative.

On December 11, the boy was again hospitalized because of respiratory distress and high-grade fever. On December 14, RT-PCR was positive for pandemic (H1N1) 2009 virus, and a 5-day regimen of oseltamivir was started. Another specimen taken the same day was negative. A high serum antibody titer (320) to pandemic (H1N1) 2009 virus was measured by hemagglutination-inhibition assay on December 16; no oseltamivir resistance mutation was found. Additional laboratory testing included a complete panel for respiratory viruses, which was negative for human metapneumovirus; respiratory syncytial virus; adenovirus; seasonal influenza virus types A and B; and parainfluenza virus types 1, 2, and 3.

These 2 cases of possible recurrent pandemic (H1N1) 2009 infection demonstrated a wide interval between illness episodes. Neither patient had accompanying immunodeficiency, and both had antibody titers far beyond the accepted seroprotective threshold for influenza (*5*), albeit ineffective. These titers probably resulted from primary infection rather than from subclinical exposure, which manifests itself as a lower titer by order of magnitude (*6**,**7*).

Virus clearance was not laboratory confirmed for patient 1 after the first episode because no samples were taken after hospital discharge. Patient 2 had both positive and negative RT-PCRs for pandemic (H1N1) 2009 virus (Table) from samples collected the same day during the second hospitalization, which also may disprove reinfection. The positive result could be explained by laboratory contamination during the RT-PCR processing that indicated a false-positive result. However, contamination is unlikely because each run of the RT-PCR was routinely accompanied by runs of negative controls (that contain water) to rule out such contamination. Nonanalytic factors such as specimen misidentification also are unlikely because the central virology laboratory, which is the national reference center, has an ISO-9000 qualification from the Standards Institution of Israel (www.sii.org.il/20-en/SII_EN.aspx). Furthermore, no other respiratory virus was found by laboratory testing at that time. The patient was infected with an oseltamivir-resistant pandemic (H1N1) 2009 virus during the first illness episode and with an oseltamivir-sensitive virus during the second episode and had 2 RT-PCRs with negative results between the episodes.

**Table:** Real-time RT-PCR for pandemic (H1N1) 2009 virus and results of experimental assay* for oseltamivir resistance mutation H275Y, Israel, 2009†

Date	Patient 1			Patient 2	
	RT-PCR	Oseltamivir resistance/sensitivity		RT-PCR	Oseltamivir resistance/sensitivity
Aug 10	Positive	Sensitive		–	–
Aug 14	–	–		Positive	Resistant
Aug 19	–	–		Positive	Resistant
Sep 1	–	–		Positive	Resistant
Sep 14	–	–		Negative	–
Nov 22	Positive	Sensitive		Negative	–
Nov 29	Negative	–		–	–
Dec 14	–	–		Positive	Sensitive
Dec 14	–	–		Negative	–
Dec 17	–	–		Negative	–
Dec 21	–	–		Negative	–

*Source (*4*). †RT-PCR, reverse transcription–PCR; –, not tested.

The novel pandemic influenza virus may be able to reinfect certain chronically ill persons. Caregivers should be aware of this trait when considering the differential diagnosis of influenza-like illness in a patient with a documented, and even treated, pandemic (H1N1) 2009 infection.
